# A Nitrate-Blind *P. putida* Strain Boosts PHA Production in a Synthetic Mixed Culture

**DOI:** 10.3389/fbioe.2020.00486

**Published:** 2020-05-25

**Authors:** Karina Hobmeier, Hannes Löwe, Stephan Liefeldt, Andreas Kremling, Katharina Pflüger-Grau

**Affiliations:** Systems Biotechnology, Technical University of Munich, Garching, Germany

**Keywords:** *Pseudomonas putida*, genetic engineering, polyhydroxyalkanoates, co-cultivation, artificial nitrogen limitation

## Abstract

One of the major challenges for the present and future generations is to find suitable substitutes for the fossil resources we rely on today. In this context, cyanobacterial carbohydrates have been discussed as an emerging renewable feedstock in industrial biotechnology for the production of fuels and chemicals. Based on this, we recently presented a synthetic bacterial co-culture for the production of medium-chain-length polyhydroxyalkanoates (PHAs) from CO_2_. This co-cultivation system is composed of two partner strains: *Synechococcus elongatus cscB* which fixes CO_2_, converts it to sucrose and exports it into the culture supernatant, and a *Pseudomonas putida* strain that metabolizes this sugar and accumulates PHAs in the cytoplasm. However, these biopolymers are preferably accumulated under conditions of nitrogen limitation, a situation difficult to achieve in a co-culture as the other partner, at best, should not perceive any limitation. In this article, we will present an approach to overcome this dilemma by uncoupling the PHA production from the presence of nitrate in the medium. This is achieved by the construction of a *P. putida* strain that is no longer able to grow with nitrate as nitrogen source -is thus nitrate blind, and able to grow with sucrose as carbon source. The deletion of the *nasT* gene encoding the response regulator of the NasS/NasT two-component system resulted in such a strain that has lost the ability use nitrate, but growth with ammonium was not affected. Subsequently, the *nasT* deletion was implemented in *P. putida cscRABY*, an efficient sucrose consuming strain. This genetic engineering approach introduced an artificial unilateral nitrogen limitation in the co-cultivation process, and the amount of PHA produced from light and CO_2_ was 8.8 fold increased to 14.8% of its CDW compared to the nitrate consuming reference strain. This nitrate blind strain, *P. putida*Δ*nasT attTn7:cscRABY*, is not only a valuable partner in the co-cultivation but additionally enables the use of other nitrate containing substrates for medium-chain-length PHA production, like for example waste-water.

## Introduction

In times of global warming, extreme weather conditions, and a growing world population, it is mandatory to dedicate arable land to food production and not “waste” it for energy formation or feedstock production for biotechnology. Along that line, efforts are directed toward replacing traditional, crop-based feedstocks like sugarcane, corn, and wheat by carbohydrates derived from ecologically more friendly sources, for example eukaryotic algae or cyanobacteria. These sources of feedstock can be produced on non-arable land with salty or brackish water. Moreover, global warming is combatted at the same time as CO_2_ is captured in bio-chemical compounds. Unfortunately, the intrinsic capacities of photosynthetic microbes to produce interesting and tailored compounds are limited and efficiencies are low.

One approach to overcome this problem is to combine the phototrophic traits of the cyanobacteria with the biotechnological abilities of a heterotrophic organism. This can be done in a synthetic mixed culture, in which the cyanobacterium produces a substrate that is simultaneously metabolized by a heterotrophic co-culture partner (Ortiz-Marquez et al., 2013; Smith and Francis, 2016; Hays et al., 2017; Löwe et al., 2017). Along that line, a number of studies were published recently, that employed a genetically engineered *Synechococcus elongatus* PCC7942 strain in a functional mixed culture (Hays and Ducat, 2015; Smith and Francis, 2016; Li et al., 2017; Löwe et al., 2017; Weiss et al., 2017). This strain, *S. elongatus cscB* carries the sucrose/H^+^-symporter CscB from *Escherichia coli* integrated into the chromosome (Ducat et al., 2012). *S. elongatus* naturally responds to elevated salt concentrations in the environment with the accumulation of sucrose as compatible solute to counteract the osmotic pressure. Thus, when the engineered strain is grown at elevated salt concentrations, sucrose is produced and exported into the medium by the activity of CscB (Ducat et al., 2012). This sugar is then taken up and converted into a valuable product by the co-culture partner, which likewise needs to be able to grow with elevated salt concentrations. Many of the defined mixed cultures were set up to produce polyhydroxybutyrate (PHB) by the heterotrophic host (Hays and Ducat, 2015; Smith and Francis, 2016; Weiss et al., 2017). The highest productivity of 28.3 mg L^–1^ d^–1^ was reached in a mixed culture between *S. elongatus* PCC7942 *cscB* and *Halomonas boliviensis*, which compares well with PHB production by genetically engineered cyanobacteria strains (Weiss et al., 2017). We recently set up a co-cultivation for the production of polyhydroxyalkanoates (PHA) with S. *elongatus* PCC7942 *cscB* and the genetically engineered strain *P. putida*:mini-Tn*5*(*cscAB*), capable of metabolizing sucrose (Löwe et al., 2017). With this mixed culture approach a production rate of PHA of around 23.8 mg L^–1^ d^–1^ was reached under nitrogen limiting conditions. However, at the end of the process a major fraction of sucrose was left untouched by *P. putida cscAB*. To face this problem, we recently engineered a more efficient sucrose consuming strain, *P. putida EM178 attTn7:cscRABY*, by the introduction of the gene cluster for sucrose metabolism from *Pseudomonas protegens* Pf-5 (Löwe et al., 2020). This strain was able to grow on sucrose with growth rates comparable to the ones obtained with the monomers glucose and fructose. *P. putida* is a very suitable partner for co-cultivations as it combines various traits, including its genetic tractability and its general stress resistance (Nikel et al., 2016), which is of great importance when grown in the non-optimal environment of the photobioreactor with elevated salt concentrations.

Natural polymers like PHA that show thermoplastic, polypropylene-like properties, could be a valuable substitute for conventional petroleum-based plastic. Depending on the chain-length of the 3-hydroxyalkanoic acids, the polymers have different mechanical properties: Most bacteria produce short chain-length PHA (scl-PHA) which consist mainly of 3-hydroxybutyrate monomers and have limited mechanical properties (Wecker et al., 2015) as they tend to be brittle when not combined with other 3-hydroxyalkanoic acids (Muhammadi et al., 2015). Only a few genera can produce longer chain-length PHAs that are more interesting for applications due to their superior and more flexible properties (Jiang et al., 2012; Wecker et al., 2015; Fontaine et al., 2017). *P. putida* is one of these native producers of medium chain-length PHA (mcl-PHA), either from lipid based substrates or carbohydrates (Huijberts et al., 1992) in conditions of one or multiple nutrient starvation (Poblete-Castro et al., 2012). *P. putida* can accumulate around 20% of its cellular dry weight as PHAs in conditions of carbon surplus and nitrogen limitation (Huijberts et al., 1992; Poblete-Castro et al., 2014). However, this is a situation difficult to achieve in a co-culture, as the other partner –at best, should not perceive any limitation.

The common growth medium for *S. elongatus* is the BG-11 medium (ATCC Medium 616) for blue-green algae (Stanier et al., 1971), which provides nitrate as nitrogen source. The modified BG-11^+^ medium we adapted for the co-cultivation of *S. elongatus* and *P. putida* consequently also has nitrate as nitrogen source (Löwe et al., 2017). In bacteria the assimilation of nitrate takes place by the reduction of nitrate to nitrite and in a second step to ammonium by the activity of nitrate reductase and nitrite reductase. In *P. putida* the availability of nitrate or nitrite is detected by a two-component sensory system consisting of the sensor NasS and the response regulator NasT (Caballero et al., 2005; Luque-Almagro et al., 2013; Supplementary Figure S1). In the presence of nitrate or nitrite the sensory protein NasS (encoded by PP_2093) binds the substrate, dissociates from the stable complex with NasS and NasT (encoded by PP_2094) is released. The unbound NasT protein then activates transcription of the assimilating nitrate and nitrite reductases encoded by PP_1703 and *nirBD* (PP_1705, PP_1706), responsible for the reduction of nitrate to ammonium. Furthermore, it was shown that the transcription of *nasT* is highly induced right at the beginning of NH_4_^+^ deficiencies, suggesting that it forms part of the early response to ammonium depletion and helps the cell in preparing for the immediate consumption of the alternative nitrogen source nitrate (Mozejko-Ciesielska et al., 2017).

In this work, we will present a metabolic engineering approach to uncouple the PHA production by *P. putida* from the presence of nitrate in the medium. This is achieved by the development a “nitrate blind” mutant, which allows the introduction of an artificial unilateral nitrogen limitation in the co-cultivation process. This enabled us not only to increase the PHA produced from CO_2_ and light to 14.8% of the CDW compared to <1% in the reference strain, but additionally opens up the use of other nitrate containing substrates, like waste-water, for medium-chain-length PHA production with *P. putida*.

## Results and Discussion

### NasT Is Essential for the Metabolism of Nitrate by *P. putida*

**FIGURE 1:**
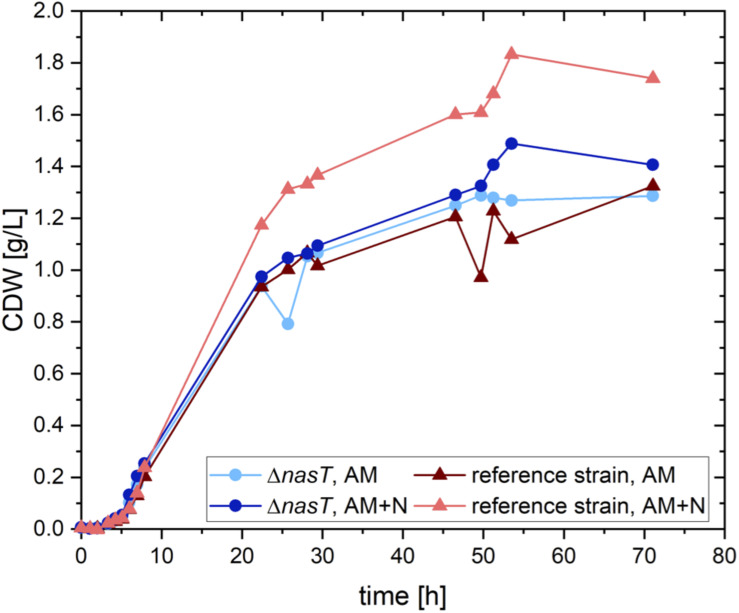
Growth of *P. putida EM178* Δ*nasT* with NH_4_Cl is not affected. Shown is the calculated cellular dry weight (CDW) for *P. putida* EM178 (red triangles) and *P. putida EM178* Δ*nasT* (blue circles) grown on glucose in M9 minimal medium with either ammonium as sole nitrogen source (NH_4_Cl 0.22 g/L) or with a mixture of ammonium and nitrate (NH_4_Cl 0.22 g/L + NaNO_3_ 0.10 g/L). The additional growth of *P. putida* EM178 compared to the other strains can be attributed to the nitrate in the medium available as nitrogen source for this strain, but not for *P. putida EM178* Δ*nasT.* Shown are the results of one representative experiment.

In order to uncouple the accumulation of PHA by *P. putida* from the presence of nitrate, we aimed to construct a nitrate-blind strain. As a target system we chose the nitrate/nitrite sensing two-component system NasS/NasT (Supplementary Figure S1). To this end, a clean deletion of *nasT* was introduced into *P. putida EM178*, a prophage free derivative of *P. putida* KT2440 by I-SceI aided double homologous recombination (Martínez-García and de Lorenzo, 2011). The resulting strain *P. putida EM178* Δ*nasT* was no longer able to grow with nitrate as sole nitrogen source (Supplementary Figure S2). However, the growth with ammonium was not affected (Figure 1). Both strains showed a similar growth behavior in the presence of ammonium as sole nitrogen source and reached final cellular dry weights of around 1.3 g/L. To test whether the addition of nitrate, a situation found in the co-cultivation, has an effect on the recombinant strain *P. putida EM178* Δ*nasT*, both strains were grown with ammonium and nitrate (see Supplementary Table S1). The recombinant strain *P. putida EM178* Δ*nasT* showed a growth behavior comparable to the one observed in the absence of nitrate, indicating that the additional presence of nitrate had no negative effect on *P. putida* EM178 Δ*nasT.* The parental strain *P. putida* EM178, however, grew best in the presence of both nitrogen sources up to around 1.75 g/L CDW. The overall amount of nitrogen was increased 1.3 fold by the addition of nitrate, which generates a 1.35 fold increase in the final CDW reached by *P. putida* EM178. The growth rates were similar in all cases, ranging between 0.52 and 0.55 h^–1^ (Table 1). Thus, the Δ*nasT* strain grew comparably to the parental strain with ammonium as nitrogen source and was not influenced by the presence of nitrate, as it is unable to induce the nitrate assimilatory pathways. This effect of *nasT* disruption was already observed in the close relative *Pseudomonas aeruginosa* PAO1 (Romeo et al., 2012).

**TABLE 1:** Growth rates of *P. putida EM178* and *P. putida EM178 ΔnasT on glucose*.

	**Growth rates [h^–1^] ± SD^a^**	
	
	**AM**	**AM + N**
*P. putida EM178*	0.517 ± 0.015	0.528 ± 0.017
*P. putida EM178* Δ*nasT*	0.54 ± 0.03	0.55 ± 0.03

**P. putida* strains were grown in M9 medium with ammonium (AM) (0.22 g/L NH_4_Cl) and with or without the addition of nitrate (N) (0.1 g/L NaNO_3_). Shown are the means of biological duplicates and the standard deviation. ^*a*^Standard deviations were determined by propagation of error of the uncertainties of two independent biological replicates.*

### Construction of a Nitrate-Blind Sucrose Metabolizing *P. putida* Suited for the Co-culture

However, to employ the nitrate-blind mutant strain in the co-cultivation, it has to be able to metabolize sucrose, a trait not intrinsically present in *P. putida* (Nogales et al., 2019). Therefore, the deletion of *nasT* was next introduced into the genetically engineered *P. putida EM178 attTn7:cscRABY*, which is capable to grow on sucrose as sole carbon source (Löwe et al., 2020), yielding *P. putida EM178 attTn7:cscRABY* Δ*nasT*. For the sake of simplicity, the strain *P. putida EM178 attTn7:cscRABY* will from here on be referred to as “*P. putida cscRABY”* and *P. putida EM178 attTn7:cscRABY* Δ*nasT* as “*P. putida cscRABY* Δ*nasT”* (see Supplementary Table S2 for more detail).

**FIGURE 2:**
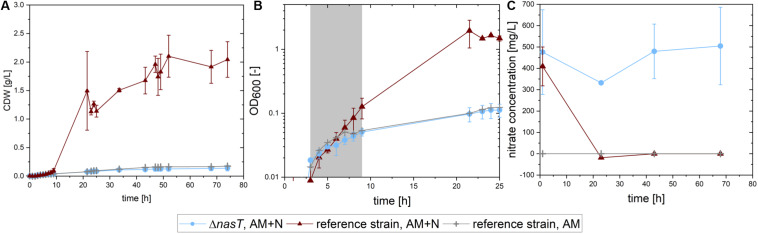
Growth of *P. putida cscRABY and P. putida cscRABY* Δ*nasT* on sucrose. *P. putida cscRABY* (red triangles) and *P. putida cscRABY* Δ*nasT* (blue circles) were grown in M9 medium with low ammonium concentrations (AM = 0.03 g/L NH_4_Cl) and with or without the addition of nitrate (N = 1 g/L NaNO_3_) to simulate the conditions of the co-cultivation. **(A)** The development of the cellular dry weight (CDW) during the cultivation. CDW was calculated using an OD/CDW correlation (see Supplementary Figure S3). **(B)** The optical density measured at 600 nm along growth. The growth rate μ (see Table 2) was determined in the exponential growth phase (gray shaded area). **(C)** The nitrate concentration in the supernatant of the cultures.

First, we analyzed the growth of both strains on sucrose with low ammonium concentrations and an excess of nitrate, to simulate the conditions in the co-cultivation. As control for the situation as it should be perceived by *P. putida cscRABY* Δ*nasT*, which can only grow on the ammonium present in the medium, *P. putida cscRABY* was grown solely with the low ammonium concentration. The growth rate, the biomass produced, and the nitrate consumed were compared (Figure 2). *P. putida cscRABY* grew to a CDW of 2.04 ± 0.313 g/L in the medium containing both nitrogen sources, whereas the *nasT* mutant, reached a biomass concentration of only about 0.14 g/L, even though nitrate was readily available (Figure 2C), corroborating the results from above. In fact, in the control experiment with *P. putida cscRABY* with ammonium only a similar CDW of about 0.18 g/L was reached.

**TABLE 2:** Growth rates of *P. putida cscRABY* and *P. putida cscRABY* Δ*nasT* on sucrose.

	**Growth rates [h^–1^] ± SD**	
	
	**AM**	**AM + N**
*P. putida cscRABY*	0.20 ± 0.04^b^	0.39 ± 0.08^c^
*P. putida cscRABY* Δ*nasT*	n.d.^a^	0.162 ± 0.008^c^

**P. putida* strains were grown in M9 medium with low ammonium (AM) concentrations (0.03 g/L NH_4_CL) and with or without an excess of nitrate (N) (1 g/L NaNO_3_). Shown are the means of biological duplicates and the standard deviation. ^*a*^Not determined. ^*b*^The standard deviation was derived from the error of regression. ^*c*^The standard deviation was derived from two independent biological replicates.*

This is also reflected in the growth rates (Figure 2B and Table 2): A similar growth rate was reached by the Δ*nasT* strain with both nitrogen sources and *P. putida cscRABY* grown on ammonium only. When *P. putida cscRABY* is grown with ammonium and nitrate, a higher growth rate is reached, a consequence of a simultaneous consumption of both nitrogen sources by the culture.

To confirm that *P. putida cscRABY* Δ*nasT* was no longer able to take up nitrate, the nitrate concentration was measured along growth (Figure 2C). *P. putida cscRABY* depleted nitrate in the first 20 h, whereas in the culture with *P. putida cscRABY* Δ*nasT* nitrate remained detectable for the time measured. Thus, the disruption of the two-component sensing system NasT/NasS by deletion of *nasT* in the sucrose consuming *P. putida cscRABY* generated a strain which is blind to nitrate in conditions resembling the situation found in the co-cultivation.

### Co-cultivation of *P. putida* Δ*nasT* and *S. elongatus cscB* for PHA Production

The next step was to test the potential of *P. putida cscRABY* Δ*nasT* in the co-culture with *S. elongatus cscB* to produce PHA from light and CO_2_. To provide proof of concept and to allow for parallel cultivations under comparable conditions, we performed the co-cultivation experiments in shaking flasks under constant illumination. All co-cultivations started with an exclusively auxotrophic growth phase of *S. elongatus cscB* for 3 days with solely nitrate as nitrogen source. Then the inoculation with *P. putida cscRABY* Δ*nasT* and simultaneous addition of ammonium took place. IPTG and elevated salt concentration were present from the beginning to induce sucrose production and excretion.

**FIGURE 3:**
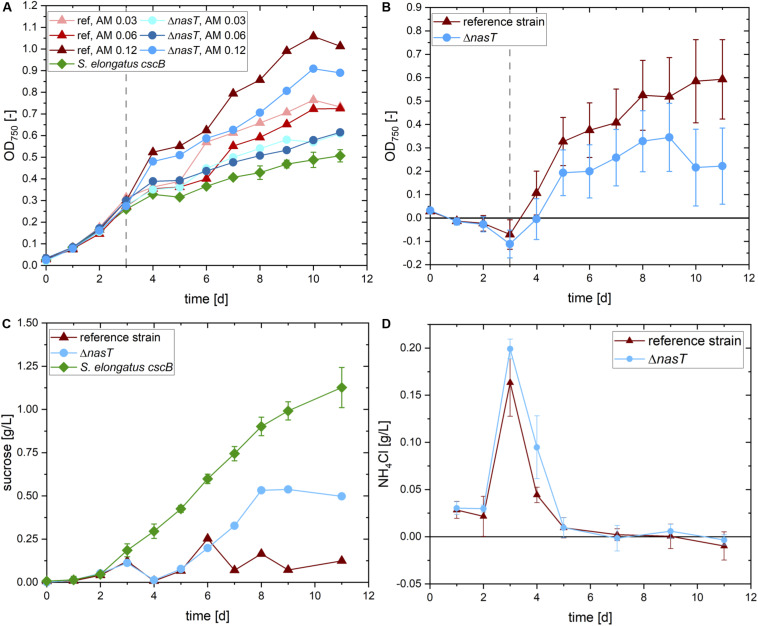
Co-cultivation of *S. elongatus cscB* and *P. putida cscRABY* Δ*nasT*. **(A)** Total OD at 750 nm of the cultures with different ammonium concentrations. *S. elongatus cscB* (green diamonds) was grown in mono-culture as control. Co-cultivations were done with *P. putida cscRABY* (ref; triangles) or with *P. putida cscRABY* Δ*nasT* (Δ*nasT*; circles), AM 0.03: 0.03 g/L NH_4_Cl; AM 0.06: 0.06 g/L NH_4_Cl; AM 0.12: 0.12 g/L NH_4_Cl. **(B)** Estimated optical density of the *P. putida* fraction of the co-cultivations with 0.12 g/L ammonium of *P. putida cscRABY* (red triangles) and *P. putida cscRABY* Δ*nasT* (blue circles). **(C)** Sucrose present in the control cultivation with *S. elongatus cscB* alone (green squares), or the co-cultivations with 0.12 g/L ammonium with *P. putida cscRABY* reference strain (red triangles), or *P. putida cscRABY* Δ*nasT* (blue circles). **(D)** Development of the NH_4_Cl concentrations in the co-cultivations with 0.12 g/L ammonium with *P. putida cscRABY* reference strain (red triangles) or *P. putida cscRABY* Δ*nasT* (blue circles).

In a first round of experiments, we aimed to find the optimal ammonium concentration to allow for sufficient initial biomass formation of *P. putida cscRABY* Δ*nasT*. The availability of ammonium for *P. putida* cannot be predicted as it is likewise metabolized by *S. elongatus cscB.* An overview of the experimental setup is given in Supplementary Figure S4 and the results are depicted in Figure 3A. Three different ammonium concentrations were tested in the co-cultivation of *S. elongatus cscB* with both, *P. putida cscRABY* Δ*nasT* and as control with *P. putida cscRABY*. To get an estimation of the biomass and sucrose produced by *S. elongatus cscB*, this strain was also grown in mono-culture. In the first auxotrophic phase before the inoculation with the heterotrophic co-culture partner and the addition of different ammonium concentrations, all eight replicates of *S. elongatus cscB* showed a uniform behavior (Figure 3A). After inoculation with *P. putida* a clear difference in the optical density (OD) can be observed in the different setups. In every case, the addition of *P. putida cscRABY* Δ*nasT* and ammonium led to higher optical densities compared to the mono-cultures of *S. elongatus cscB* without ammonium. This can be explained by the introduction of additional cells, but also by the addition of ammonium, which promotes growth of the cyanobacterium as well. Comparing the co-cultivations with the Δ*nasT* mutant to the ones with *P. putida cscRABY* reveals, that in all cases a better overall growth is observed with *P. putida cscRABY*. As for this strain nitrate is available as nitrogen source, it reached higher optical densities that contribute to the total OD of the co-culture. A comparison of the development of the total OD with different ammonium concentrations in each of the co-cultivations revealed that no clear difference was observed with 0.03 g/L or 0.06 g/L NH_4_Cl, i.e., that although the ammonium concentration was doubled the total OD did not increase correspondingly. Therefore, it was assumed that theses concentrations were too low to allow for substantial growth of *P. putida*. The addition of 0.12 g/L NH_4_Cl, however, led to an increase in the overall OD, compared to the lower NH_4_Cl concentrations, suggesting that ammonium was available for growth of *P. putida*. Thus, this co-culture was analyzed in more detail.

To get a rough estimation of the proportion of the OD achieved by *P. putida* cells, the signal stemming from *P. putida* was traced back using its different absorption characteristics (for details see Experimental Procedures and Supplementary Material). Therefore, a technique was applied similar to the fluorescence based “spectral unmixing” method described by Lichten et al. (2014). Instead of using variations in fluorescence intensity, here we used the different absorption characteristics of both bacteria. In particular, *S. elongatus* absorbs light via some pigments involved in photosynthesis, like chlorophyll and carotenoids, in a very different way than *P. putida*. By measuring at wavelengths that are specific and unspecific for each of the co-culture partners, information on the quantity of both can be obtained. This allowed us to calculate the proportion of *P. putida* of the total OD. The estimated optical densities reached by *P. putida cscRABY* or *P. putida cscRABY* Δ*nasT* are shown in Figure 3B. At all time points, the estimated OD of *P. putida cscRABY* exceeds the one estimated for the Δ*nasT* mutant. Furthermore, according to these estimations, *P. putida cscRABY* makes up a higher proportion of the overall OD than the Δ*nasT* strain, which can be explained by the competition with *S. elongatus cscB* for nitrate.

Every day the sucrose content of these cultivations was determined by means of HPLC (Figure 3C). *S. elongatus cscB* excreted sucrose into the medium at a rate of 0.14 ± 0.014 g/L d and a titer of 1.13 ± 0.117 g/L was reached after 11 days. To bring this into context, this is roughly half of what was obtained in the bioreactor, where the cells are optimally supplied with light and CO_2_ (Löwe et al., 2017). In both co-cultivations sucrose concentration decreased to the detection limit 1 h after the inoculation with the *P. putida* partner. However, whereas in the co-cultivation with *P. putida cscRABY* the sucrose concentration stayed on a low level throughout the experiment, it increased in the co-cultivation with the Δ*nasT* mutant strain to around 0.5 g/L of sucrose at the end of the experiment. This suggests that in the co-cultivation with *P. putida cscRABY* the sucrose produced by *S. elongatus cscB* is simultaneously taken up and metabolized by the heterotroph. In the co-cultivation with the Δ*nasT* mutant in contrast, the sucrose consumption pattern diverged from the one obtained with *P. putida cscRABY* from day six on. Measuring the NH_4_Cl concentration revealed that this is the time when NH_4_Cl becomes scarce and was no longer detectable from day seven on (Figure 3D). This suggests that the Δ*nasT* mutant stopped growing when ammonium was depleted. As a consequence, the Δ*nasT* mutant reduced the uptake of sucrose, whereas *P. putida cscRABY* continued to grow with nitrate as nitrogen source.

The plateau in the sucrose concentration reached at day eight might have different explanations on which only can be speculated. It could reflect the moment when cells switched to increased PHA accumulation which comes along with increased uptake of sucrose. However, it can also not be excluded that sucrose production by the cyanobacterium ceased for another reason. Nevertheless, the conditions from day seven on in the co-cultivation with *P. putida cscRABY* Δ*nasT*, where no nitrogen source is available for the heterotroph, but the carbon source is still present, should resemble a situation in which PHA accumulation is promoted.

The amount of PHA produced by the respective strain at the end of the co-cultivation experiment was determined. Indeed, *P. putida cscRABY* Δ*nasT* accumulated 25.24 mg/L PHA, which make up 14.8% of its calculated dry weight. In contrast, in *P. putida cscRABY* less than 1% of its calculated dry weight corresponded to PHAs (2.86 mg/L). Thus, deletion of *nasT* led to a 8.8 fold increase in the PHA accumulation in conditions in which nitrate is present. The distribution pattern of 3-hydroxyalkanoic acids is typical for *P. putida* (Supplementary Table S3), with 3-hydroxydecanoic acid being the most abundant monomer and does not differ substantially from the one usually obtained in *P. putida* and also reported in earlier co-cultivations in a photobioreactor (Poblete-Castro et al., 2014; Löwe et al., 2017).

Thus, by disruption of the nitrate sensing TCS, the PHA accumulation is uncoupled from the presence of nitrate in the medium. As a result, this strain experiences nitrogen limitation considerably earlier during the co-cultivation than *P. putida cscRABY* as it cannot sense nitrate and in consequence does not induce the assimilatory proteins. The lack of nitrogen assimilation should result in an internal decrease of glutamine, which triggers accumulation of the storage compound PHA. This strain is a promising candidate for PHA production from CO_2_ and light not only at larger scale, but also opens up the use of other nitrate containing substrates, like waste-water, for PHA production.

This co-culture setup combined two novel elements on the side of the heterotroph compared to the published co-cultivations: (i) the blindness to nitrate, which uncouples PHA accumulation from the availability of nitrate and allows us to run the fermentation under normal nitrogen conditions, and (ii) the higher efficiency of sucrose consumption of *P. putida cscRABY* (Löwe et al., 2020), which represents a clear advance in the co-culture platform for PHA production with *P. putida*.

### Stabilizing Effect of Co-cultivation of *P. putida* and *S. elongatus cscB*

**FIGURE 4:**
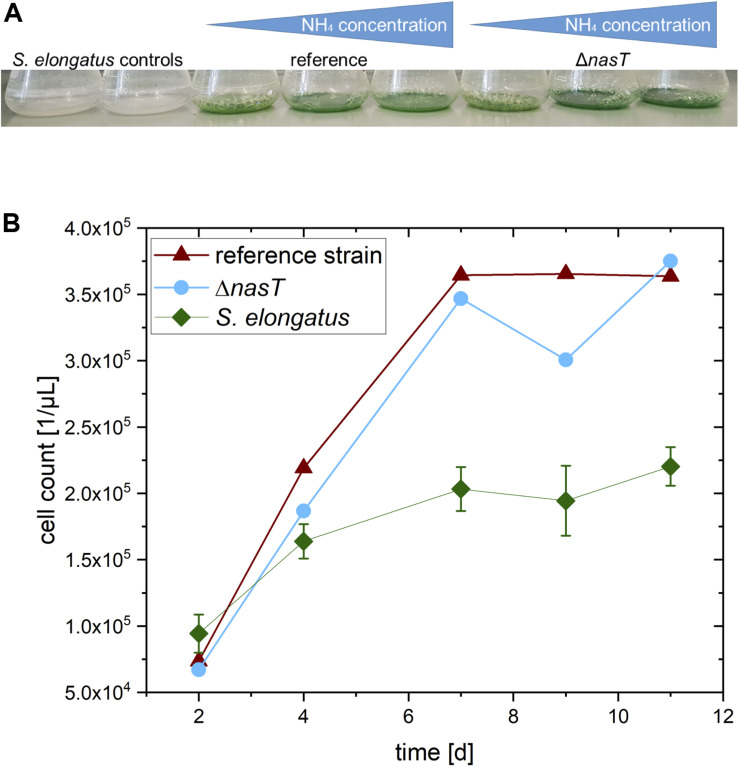
Stabilizing effect of the co-cultivation. **(A)** Color of the cultures 4 weeks after the end of the experiment. Note that the mono-cultures have experienced complete chlorosis, whereas the co-cultures are still green. **(B)** Number of *S. elongatus cscB* cells in the different setups: *S. elongatus*, control: *S. elongatus cscB* in mono-culture; reference strain: Co-cultivation of *S. elongatus cscB* with *P. putida cscRABY* reference strain; Δ*nasT*: Co-cultivation of *S. elongatus cscB* with *P. putida ΔnasT*.

At the end of the co-cultivation experiment described above, we observed that the two *S. elongatus* monocultures started to lose the green pigmentation, whereas the co-cultures did not. This was even more pronounced 4 weeks after inoculation (Figure 4A). Furthermore, the intensity of the green color of the cultures increased with increasing ammonium concentration. The loss of pigmentation in cyanobacteria is known as chlorosis, a process that describes the depigmentation of cyanobacteria due to degradation of chlorophyll (Forchhammer and Schwarz, 2019). The fitness and viability of a cyanobacterial culture can be estimated from their chlorophyll content, which is reflected by the characteristic green color. When they are stressed or starved, they respond with chlorosis.

This observation that the presence of *P. putida* seems to have a stabilizing effect on the cyanobacterium was strengthened by the development of the cyanobacterial cell number in the co-cultivation (Figure 4B). In the mono-cultures of *S. elongatus cscB* a final cell count of around 2.2 × 10^5^ cells/μL was determined, whereas in the co-cultivation setups *S. elongatus cscB* reached over 3.6 × 10^5^ cells/μL. This effect was independent of the specific *P. putida* strain. However, the exact reason for the chlorosis cannot be determined from the experiment. It might be the consequence of the presence of *P. putida* cells. Thus, it can be speculated that the depletion of oxygen by respiration of *P. putida* provides an advantage for the cyanobacteria. On the other hand, the effect can also be attributed to the addition of ammonium together with the inoculation of *P. putida*, which might provide an additional advantage for the cyanobacteria. Another possible positive effect on *S. elongatus cscB* might be the depletion of sucrose in the culture medium by the activity of *P. putida.* Nevertheless, any combination thereof is also possible and will be in the focus of another study in out laboratory.

The stabilizing effect of co-cultivations on one of the co-culture partners was not only observed by us, but has been previously reported also by other (Hays et al., 2017). Thus, co-cultivations do not only provide an advantage over mono-cultures due to the division of labor allowing for functions that are difficult to program in individual cells, but also might have an effect on the stability of the system.

## Conclusion

Biopolymers like PHA are often accumulated in conditions of nutrient depletion, a situation which sometimes is difficult to achieve in a biotechnological application, or at least requires considerable efforts. Genetic engineering and process engineering open up ways to mimic limitations for the specific microbe, without interfering with the rest of the system. This allows not only to improve existing microbes in terms of their productivity, but may also lay the basis for the usage of novel, or less processed substrates.

Here, we reported that deletion of the *nasT* gene encoding the response regulator of the NasS/NasT two component system resulted in a strain insensitive to the presence of nitrate and unable to grow with nitrate as nitrogen source. Nevertheless, growth but with other nitrogen sources, like ammonium, remained unaffected. The introduction of this deletion into the sucrose consuming *P. putida cscRABY* created a strain very well suited as PHA producer strain in the co-cultivation with *S. elongatus cscB.* As in this strain the PHA accumulation was uncoupled from the presence of nitrate it was not necessary to apply a nitrate limitation on the co-cultivation to induce a regime in *P. putida* that allowed for accumulation of the polymer. The final PHA titer reached by this recombinant strain was about 9-fold higher than in *P. putida cscRABY*. However, these numbers are based on the snapshot at the end of the experiment. To quantify the improvement of this tailored strain, next we will determine the PHA production rate in an optimal environment, i.e., the photobioreactor, where controlled conditions can be ensured. Apart from being used as co-cultivation partner, this strain can also be used for mcl-PHA production from other feedstocks as nitrate containing waste and surplus material. The engineering strategy applied in this work represents an important step toward mcl-PHA production from carbohydrates, which have great potential as a source of sustainable bioplastics and for medical applications (Kniewel et al., 2019) as well as for the production of chiral 3-hydroxy fatty acids that themselves can serve as precursors for various high-value products (Lee et al., 1999).

## Materials and Methods

### Bacterial Strains and Plasmids

*Escherichia coli* DH5αλ-pir was used for the extraction of plasmids, transformation and as the plasmid donor in conjugation. *E. coli* HB101 (pRK600) and *E. coli* DH5α (pTnS1) served as helpers in conjugation and Tn7 transposition, respectively. All *P. putida* strains used in this work are derived from *P. putida* EM178, a prophage-free derivative of *P. putida* KT2440 (created at Victor de Lorenzo’s lab at CNB, Madrid). An overview on the *P. putida* strains used in this work and how they are designated in the text is given in Supplementary Table S2. The organisms employed in the mixed culture are the autotrophic host, *S. elongatus cscB* (Ducat et al., 2012) and *P. putida* EM178 *att*Tn7:*cscRABY ΔnasT*, a derivative of *P. putida* EM178 *att*Tn7:*cscRABY* (Löwe et al., 2020), was constructed as described below.

### Bacterial Growth Conditions, Growth Experiments, and Data Analysis

*Pseudomonas putida* and *E. coli* strains were cultivated at 30°C or 37°C, respectively, in either LB or M9 mineral medium (Miller, 1974) with 2% [w/v] of either glucose for *P. putida* EM178 and *P. putida EM178* Δ*nasT*, or sucrose for *P. putida* EM178 *att*Tn7:*cscRABY* and *P. putida* EM178 *att*Tn7:*cscRABY*Δ*nasT* as carbon source.

Growth experiments with *P. putida* EM178 and *P. putida EM178* Δ*nasT* in the presence or absence of nitrate were conducted in an ammonium-reduced M9 medium: 6.77 g/L Na_2_PO_4_, 2.99 g/L KH_2_PO_4_, 0.5 g/L NaCl, and 0.22 g/L NH_4_Cl with or without the addition of 0.01 g/L NaNO_3_.

Each experiment was performed in biological duplicates. The mean growth rates were calculated by performing a linear regression in the exponential phase (first 10 h frame) and averaging the slopes for both biological replicates. The given standard deviation refers to the error between biological replicates only since the error of the linear fit was found to be negligible.

For the characterization of the sucrose-metabolizing strain (*P. putida* EM178 *att*Tn7:*cscRABY*Δ*nasT*) the ratio between ammonium and nitrate was shifted toward a higher nitrate proportion to allow only for very little growth with ammonium (0.03 g/L NH_4_CL and 1 g/L mM NaNO_3_) and 10 μg/mL Gentamicin was added to select for the integrated sucrose operon. Additionally, the trace element solution A5 originally found in BG11 medium (ATCC Medium 616) was supplemented to avoid deficiency of inorganic cations during growth on nitrate. All cultivations were carried out in 250 mL shaking flasks with 10% filling volume in a shaking incubator at 220 rpm. The precultures were initially grown in LB medium followed by a M9 medium intermediate culture (0.5 g/L NH_4_Cl). Before inoculation of the main culture the inoculum was washed once in the cultivation medium to avoid transfer of ammonium from the preculture medium.

### Co-cultivation

The co-cultivation was performed in the modified BG11^+^ medium described previously (Löwe et al., 2017) at 30°C and 120 min^–1^ rpm with 10% filling volume of 250 mL shaking flasks in the Multitron Pro incubator equipped with CO_2_ gassing and LED lighting (Infors). A photon flux density of 24 μmol m^–2^ s^–1^ was applied and air was used as the sole source of CO_2_. After inoculation with *S. elongatus cscB* an initial adaptation and auxotrophic growth phase of 3 days was allowed. Upon inoculation with *P. putida* various concentrations of NH_4_Cl were supplied in parallel: 0.03 g/L, 0.06 g/L, and 0.12 g/L to allow for initial growth of *P. putida* EM178 *att*Tn7:*cscRABY*Δ*nasT* before the PHA production phase begins. To the two control flasks without heterotrophic partner no ammonium was added. The *S. elongatus cscB* pre-cultures were grown in 100 mL shaking flasks with 40 mL BG11^+^ medium in the Multitron Pro incubator. The *P. putida* pre-cultures were initially grown in LB medium followed by a M9 medium intermediate culture. The main culture was inoculated with washed cells to eliminate carry over of nitrogen and carbon from the intermediate culture.

### Construction of *P. putida* EM178 *attTn7:cscRABY* Δ*nasT*

The gene deletion was carried out as described in Martínez-García and de Lorenzo (2011). All enzymes used were obtained from New England Biolabs (United States). The flanking regions upstream and downstream of *nasT* of 709 bp, respectively, were amplified by PCR (Q5 Polymerase). The primers used (Supplementary Table S4) for amplification introduced complementary overhangs allowing to join both fragments by overlap extension PCR (Q5 Polymerase), as well as restriction sites for the enzymes *Eco*RI and *Hin*dIII. Thus, after joining, the gene fragment was inserted into the suicide vector pSEVA212S (Silva-Rocha et al., 2013) by restriction digest and ligation (T4 DNA Ligase) to create the integration vector pSEVA212S-Δ*nasT*. This suicide vector was transferred to *P. putida* EM178 *att*Tn*:cscRABY* by triparental mating with *E. coli* HB101 (pRK600) as helper and co-integrates were selected by plating on LB with 50 mg/ml Kanamycin (de Lorenzo and Timmis, 1994). The integration was confirmed by PCR (oneTaq Polymerase) with the primers fwP_nasT_orient and rvP_nasT_seq (Supplementary Table S4), which also allowed to determine the site of the homologous recombination. Subsequently, the helper plasmid pSW-I was transferred by triparental mating with *E. coli* DH5α (pTnS1) as donor and one clone with the integrated pSEVA212S-Δ*nasT* as receptor. Expression of the *I-Sce*I endonuclease was induced with 1 mM 3-methylbenzoic acid for 4 h in a stationary culture (Martínez-García and de Lorenzo, 2011). Subsequently, different dilutions were plated on M9 solid medium with citrate as C-source (0.2% w/v), incubated at 30°C and single-colonies were examined by PCR (oneTaq Polymerase) and the correct deletion was confirmed by complete sequencing (Eurofins Genomics, Ebersberg) of the flanking regions.

### Determination of Growth, Nitrate Concentration, Ammonium Concentration, Sucrose Concentration and PHA Content

Growth of the *P. putida* cultures was followed by measuring the OD at 600 nm in a 96-well plate in the Infinite 2000 TECAN plate reader. In the case of co-cultivation with *S. elongatus cscB*, growth was followed likewise, but at 750 nm. The dry weight was approximated using an OD to dry weight correlation determined previously for the common parental strain *P. putida* EM178 (see Supplementary Figure S3).

The nitrate/nitrite concentration of culture supernatants was determined using a colorimetric assay (Nitrite/Nitrate colorimetric method, Roche Diagnostics GmbH, Penzberg, Germany) in a microplate reader based on the enzymatic reduction of nitrate to nitrite.

The ammonium concentration was determined with the Ammonia Assay (Cat. No. 11 112 732 035, Boehringer Manheim/R-Biopharm) according to the supplier’s manual.

The sucrose concentrations were measured using high performance liquid chromatography (HPLC) via a Shodex SH1011 column and the PHA content was determined by gas chromatography (GC) from 2 ml of the culture, exactly as described in Löwe et al. (2017).

### Flow Cytometry

For the cell count measurements, a 400 μL sample of the co-culture was centrifuged for 5 min at 12000 *g*. The cell pellet was then resuspended in 400 μL PBS and Nile-red resuspended in DMSO was added to a final concentration of 3.1 μg/mL. After an incubation period of 30 min at room temperature the sample was centrifuged and washed in PBS again and a 1:100 dilution was measured in the Cytoflow Flow Cytometer (Beckman Coulter). Cells were excited by a 488 nm laser and *S. elongatus cscB* could be identified by red fluorescence that is lacking in *P. putida*.

### Estimation of the Contribution of *P. putida* Cells to the OD750

The OD at 750 nm measured during the experiments is a sum of the ODs of both species.

$$O\,D_{750}=O\,D_{750,P.p\,u\,t\,i\,d\,a}+O\,D_{750,S.e\,l\,o\,n\,g\,a\,t\,u\,s}$$

For the differentiation between the OD of *S. elongatus* and *P. putida*, a technique was applied similar to the fluorescence based “spectral unmixing” method described by Lichten et al. (2014). Instead of using variations in fluorescence intensity, here we used the different absorption characteristics of both bacteria. As a photosynthetic organism, *S. elongatus* has a variety of fluorescent pigments like chlorophylls and carotenoids that strongly absorb in certain wavelength regions. In particular, we used the absorption at the wavelengths at 442 nm and 632 nm, both of which should be highly influenced by chlorophyll absorption. Control experiments with pure cultures indicated that the absorption at both wavelengths show a constant linear correlation with different correlation factors (see Supplementary Figure S5):

$$O\,D_{632}=m_{632/442}\,O\,D_{442}$$

where *m*_*632/442*_ is the correlation factor of the two absorption-values which depends on the bacterial strain.

This can be used to estimate the proportions of different bacterial species which will be shown in the following equations. First, both *P. putida* and *S. elongatus* contribute to*OD*_632_:

$$O\,D_{632}=O\,D_{632,P.p\,u\,t\,i\,d\,a}+O\,D_{632,S.e\,l\,o\,n\,g\,a\,t\,u\,s}=$$

$$=m_{632/442,P.p\,u\,t\,i\,d\,a}\,O\,D_{442,P.p\,u\,t\,i\,d\,a}+$$

$$m_{632/442,S.e\,l\,o\,n\,g\,a\,t\,u\,s}\,O\,D_{442,S.e\,l\,o\,n\,g\,a\,t\,u\,s}$$

Likewise, *OD*_*442*_is also the sum of individual absorptions of the two species:

$$O\,D_{442}=O\,D_{442,P.p\,u\,t\,i\,d\,a}+O\,D_{442,S.e\,l\,o\,n\,g\,a\,t\,u\,s}$$

Having two equations with two variables (*OD*_*442,  P.  putida*_and *OD*_*442,  S.  elongatus*_), we can solve for the single variables:

$$O\,D_{442,P.p\,u\,t\,i\,d\,a}=\frac{O\,D_{632}-m_{632/442,S.e\,l\,o\,n\,g\,a\,t\,u\,s}\,O\,D_{442}}{m_{632/442,P.p\,u\,t\,i\,d\,a}-m_{632/442,S.e\,l\,o\,n\,g\,a\,t\,u\,s}}$$

Since *OD*_*442,  P.  putida*_ also correlates linearly with *OD*_*750,  P.  putida*_ (see Supplementary Figure S6), the proportion of *P. putida* can easily be calculated from the OD at 442 nm:

$$O\,D_{750,P.p\,u\,t\,i\,d\,a}=m_{750/442}\,O\,D_{442,P.p\,u\,t\,i\,d\,a}$$

$$O\,D_{750,S.e\,l\,o\,n\,g\,a\,t\,u\,s}=O\,D_{750}-O\,D_{750,P.p\,u\,t\,i\,d\,a}$$

In total, absorption measurements at three different wavelengths are necessary to calculate these values when the correlation-factors *m*_*i/j*_ are known. The errors of the calculated, final *OD*_*750*_- values are therefore influenced not only by the errors of the correlation-factor *m*_*i/j*_, but also by the error of measurement of three ODs. We calculated the errors accordingly with Gaussian propagation of uncertainty, assuming a combined handling and instrument error of 2%.

## Data Availability Statement

All datasets generated for this study are included in the article/Supplementary Material.

## Author Contributions

KH and HL conceived and planned the experiments. KH, SL, and HL carried out the experiments. KP-G wrote the manuscript with the help of HL and KH. KP-G and AK supervised the project with the help of HL. All authors discussed the results and commented on the manuscript.

## Conflict of Interest

The authors declare that the research was conducted in the absence of any commercial or financial relationships that could be construed as a potential conflict of interest.
