# Supplementary material for: A Nitrate-Blind P. putida Strain Boosts PHA Production in a Synthetic Mixed Culture
Source: Front Bioeng Biotechnol. 2020 May 25;8:486. doi: 10.3389/fbioe.2020.00486 (PMC7261876; doi:10.3389/fbioe.2020.00486)
Supplement: Supplementary file 1 [file Data_Sheet_1.pdf]

Supplementary Material

for

A nitrate-blind *P. putida* strain boosts PHA production in a synthetic mixed culture

by

Karina Hobmeier, Hannes Löwe, Stephan Liefeldt, Andreas Kremling, Katharina Pflüger-Grau*

*** Correspondence:**
Katharina Pflüger-Grau
k.pflueger-grau@tum.de

Supplementary Table S1: Nitrogen source and concentration used in this study. Shown are the type of nitrogen source, the concentration used in the different experiments of this study, as well as the carbon source. In the first column reference is given to the respective figure.

Experiment	Strain	NH_4Cl	$NaNO_3$	C source
**Figure 1**	*P. putida* EM178, AM	0.22 g/L	---	Glucose
	P. putida EM178 *ΔnasT*, AM	0.22 g/L	---	
	P. putida EM178, AM+N	0.22 g/L	0.10 g/L	
	P. putida EM178 *ΔnasT*, AM+N	0.22 g/L	0.10 g/L	
**Figure S1**	*P. putida* EM178	---	0.10 g/L	Glucose
	P. putida EM178 *ΔnasT*	---	0.10 g/L	
**Figure 2**	*P. putida cscRABY*, AM+N	0.03 g/L	1.00 g/L	Sucrose
	P. putida cscRABY, AM	0.03 g/L	---	
	P. putida cscRABY ΔnasT, AM+N	0.03 g/L	1.00 g/L	
**Figure 3 (Co-culture)**	*S. elongatus cscB*	---	1.50 g/L	CO_2
	S. elongatus cscB<br>*P. putida cscRABY*, AM+N	0.03 g/L<br>0.06 g/L<br>0.12 g/L	1.50 g/L<br>1.50 g/L<br>1.50 g/L	CO_2<br>Sucrose
	S. elongatus cscB<br>*P. putida cscRABY ΔnasT*, AM+N	0.03 g/L<br>0.06 g/L<br>0.12 g/L	1.50 g/L<br>1.50 g/L<br>1.50 g/L	CO_2<br>Sucrose

Supplementary Table S2: Strains used in this study and how they are designated in the text. Additionally, the C-source used for the respective strain in this study is shown.

Strains	Designation	C source	Reference
S. elongatus cscB	*S. elongatus cscB*	CO_2	(Ducat et al., 2012)
P. putida EM178	*P. putida* EM178	Glucose	Obtained from Víctor de Lorenzo
P. putida EM178 *ΔnasT*	*P. putida* EM178 *ΔnasT*	Glucose	this work
P. putida EM178 attTn7::*cscRAB-scrY*	*P. putida cscRABY*	Sucrose	(Löwe et al., 2018)
P. putida EM178 attTn7::*cscRAB-scrY ΔnasT*	*P. putida cscRABY ΔnasT*	Sucrose	this work

Supplementary Table S3: Distribution of chain-lengths per mass fraction in PHA produced by *P. putida cscRABY ΔnasT* in the mixed culture. As comparison the distribution of chain-length from the co-cultivation under nitrate limiting conditions is shown (Löwe et al., 2017).

**Chain length (carbon number)**	**Mass fraction (%)**	
	This study	Löwe et al., 2017
6	0	4.2
8	23.2	25.2
10	62.4	58.4
12	6.0	4.4
12:1	8.4	7.8

Supplementary Table S4: Oligonucleotides used in this study. Restriction sites are shown in minuscules and homologous regions for recombination are underlined.

**Oligo name**	**Sequence 5' -> 3'**	**Function**
nasT ko fw1	TTTTgaattcCGGGATCAGCGCAAAGCCGAAC	Amplification of the upstream fragment for the construction of pSEVA212S-*ΔnasT*
nasT ko rv1	GTGAGGGGGACGAACTGCGCTGAAAATTCCGAGCAAGC	
nasT ko fw2	GGAATTTTCAGCGCAGTTCGTCCCCCTCACGCGCCGACAC	Amplification of the downstream fragment for the construction of pSEVA212S-*ΔnasT*
nasT ko rv2	AAAAaagcttTGGTGCCACCGGCGCAGATG	
fwP_nasT_orient	CGCCAGGGTTTTCCCAGTCAC	Identification of recombination site
fwP_nasT_seq	ATGAACCACCAGCTGTCACG	PCR and sequencing primers
rvP_nasT_seq	TGACCAACCCTGAGGCACTG	

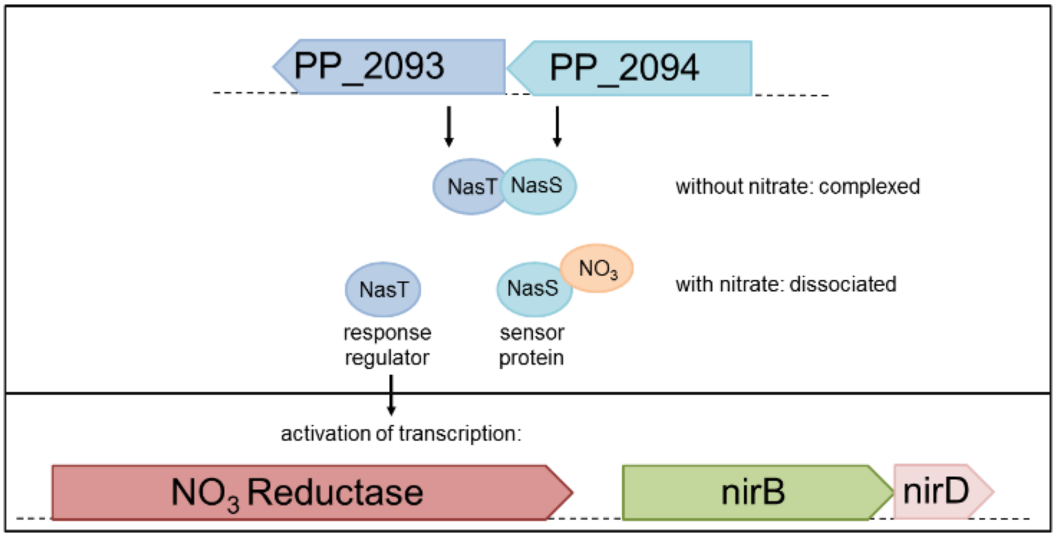

Supplementary Figure S1. Nitrate sensing NasT/NasS two-component system of *P. putida*. The availability of nitrate or nitrite is detected by the sensory protein NasS (encoded by PP_2093), which forms the stable complex with NasS (encoded by PP_2094). Upon substrate binding, NasS dissociates from the complex and NasT is released. The unbound NasT protein then activates transcription of the assimilating nitrate reductase, encoded by PP_1703 and the nitrite reductases encoded *nirBD* (PP_1705, PP_1706), responsible for the reduction of nitrate to ammonium.

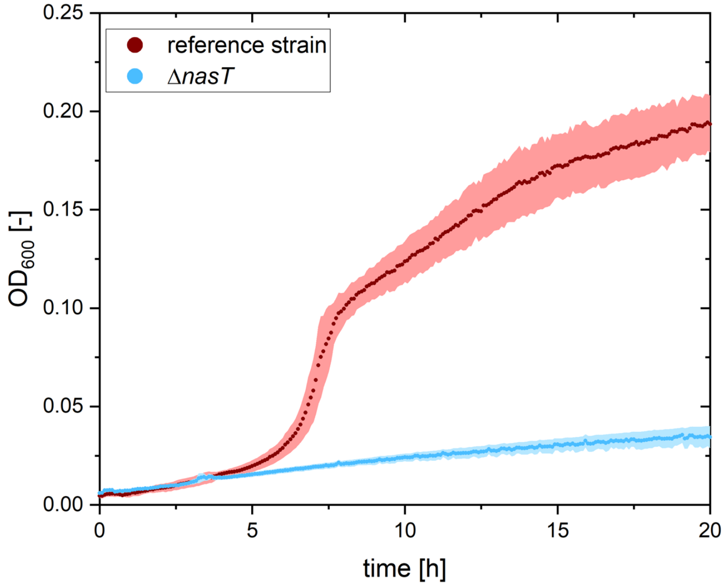

Supplementary Figure S2. *P. putida ΔnasT* cannot grow with $NaNO_3$ as sole nitrogen source. Shown is the optical density measured at 600 nm for *P. putida* EM178 (red) and *P. putida* EM178 *ΔnasT* (blue) grown on glucose in M9 minimal medium with $NaNO_3$ as sole N-source in a 96-well plate. The small linear increase in optical density in the culture of *P. putida ΔnasT* can be attributed a carry-over of NH_4Cl from the preculture during inoculation (in the first 3 hours) and to evaporation from the wells of the 96-well plate incubated at 30°C. The lines represent the mean of 3 replicates and the shaded area the standard deviation.

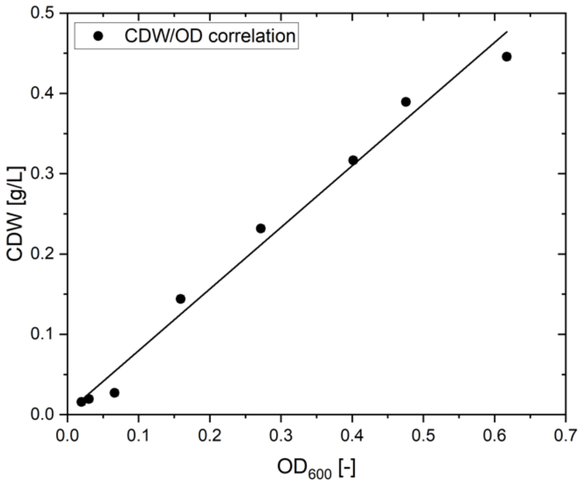

Supplementary Figure S3. Correlation of CDW and optical densities OD_{600} for *P. putida* EM178. Data was obtained from three biological replicates of *P. putida* EM178 grown in M9 minimal medium with glucose as the sole carbon source in shaking flasks. The plots were fitted using linear regression to determine the correlation coefficient.

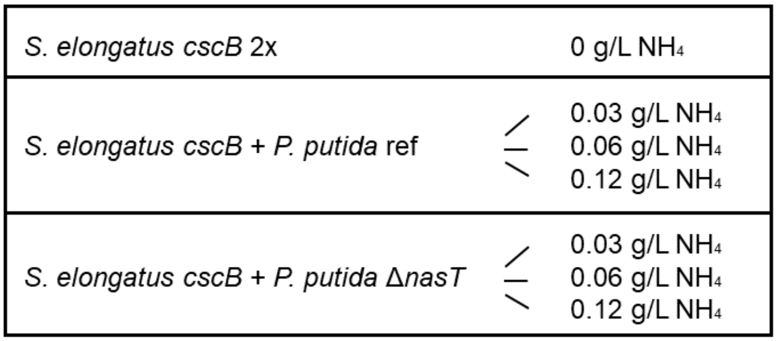

Supplementary Figure S4. Experimental setup of the co-cultivation experiments. As control two mono-cultures of *S. elongatus cscB* were conducted. Three co-cultivations were performed with *P. putida cscRABY ΔnasT* and *P. putida cscRABY* (ref) with varying ammonium concentrations as indicated.

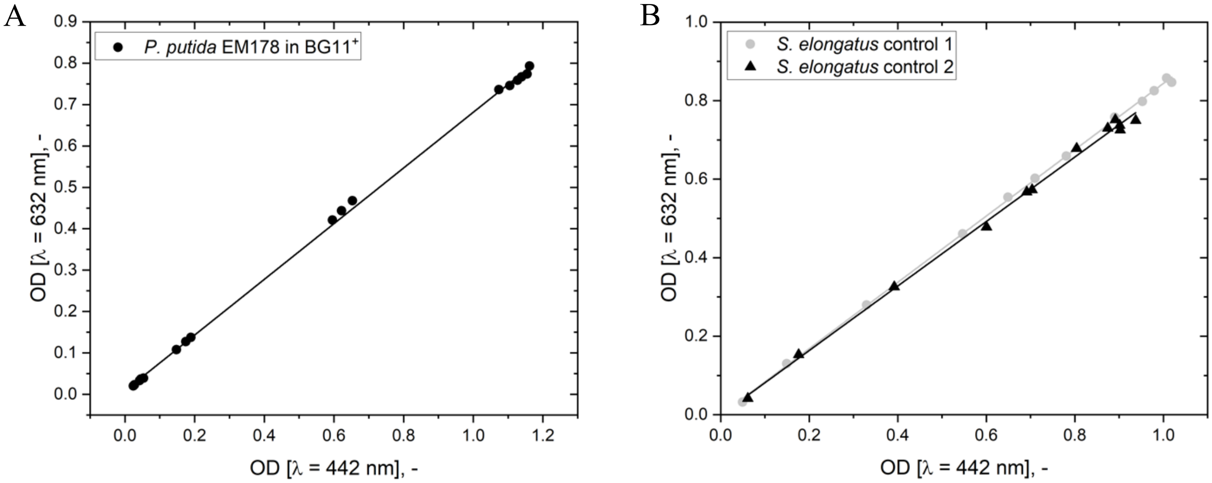

Supplementary Figure S5. Correlation of optical densities (OD) measured at wavelengths of 442 nm and 632 nm for *P. putida* EM178 (A) and *S. elongatus cscB* (B). *P. putida* was cultured in $BG11^+$ medium with a mixture of glucose and fructose, 1.5 g/L each, as carbon source in shaking flasks. The data for *S. elongatus cscB* was obtained from two replicates as part of the mixed culture experiments. The plots were fitted using linear regression to determine the correlation coefficients.

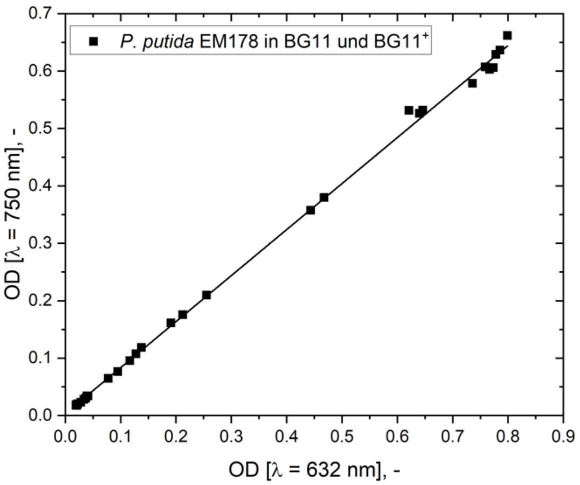

Supplementary Figure S6. Correlations of optical densities (OD) measured at wavelengths of 632 nm and 750 nm for *P. putida* EM178. Data was obtained from five biological replicates of *P. putida* EM178 grown as mono-culture in BG11 (2 replicates) and $BG11^+$ medium (3 replicates) with glucose and fructose 1.5 g/L each as carbon source. The plots were fitted using linear regression to determine the correlation coefficient.

Materials and Methods

Growth on nitrate in 96-well plate (Fig. S2)

A 96-well plate screening in M9-based medium containing 0.1g/L $NaNO_3$ as sole N-source (6.77 g/L Na_2HPO_4, 2.99 g/L KH_2PO_4, 0.5 g/L NaCl, 0.1 g/L $NaNO_3$, 0.24 g/L $MgSO_4$, 20 g/L Glucose) was performed. Initially, a single colony was picked of either strain, *P. putida* EM178 or *P. putida* EM178 *ΔnasT,* and grown in LB medium. This was done in biological triplicates. Subsequently, an intermediate culture in M9 medium with 1 g/L NH_4Cl as N-source and 5 g/L glucose as C-source was inoculated using an aliquot of the LB precultures. When these cultures reached the exponential growth phase, an aliquot was transferred to the main culture with only $NaNO_3$ to an initial OD of 0.01 in a 96-well plate with a volume of 0.2 mL. The cultures were incubated in an automated microplate reader (Tecan) at 30 °C and the optical density at 600 nm was followed for the next 20 hours. The whole experiment was conducted in biological triplicates and the mean and standard deviation were calculated.

Literature

Ducat, D. C., Avelar-Rivas, J. A., Way, J. C., and Silver, P. A. (2012). Rerouting carbon flux to enhance photosynthetic productivity. *Appl Environ Microbiol* 78, 2660–2668. doi:10.1128/AEM.07901-11.

Löwe, H., Schmauder, L., Hobmeier, K., Kremling, A., and Pflüger-Grau, K. (2017). Metabolic engineering to expand the substrate spectrum of *Pseudomonas putida* toward sucrose. *Microbiologyopen* 6. doi:10.1002/mbo3.473.

Löwe, H., Sinner, P., Kremling, A., and Pflüger-Grau, K. (2018). Engineering sucrose metabolism in *Pseudomonas putida* highlights the importance of porins. *Microb Biotechnol*. doi:10.1111/1751-7915.13283.
